# Bridging therapy for oral anticoagulation increases the risk for bleeding-related complications in total joint arthroplasty

**DOI:** 10.1186/s13018-015-0285-6

**Published:** 2015-09-17

**Authors:** Martijn Haighton, Diederik H R Kempen, Nienke Wolterbeek, Louis N. Marting, Martijn van Dijk, Remmelt M R Veen

**Affiliations:** Department of Orthopaedic Surgery, St. Antonius Hospital, P.O. Box 2500, 3430 EM Utrecht, The Netherlands; Department of Orthopaedic Surgery, OLVG Hospital, Oosterpark 9, 1091 AC Amsterdam, The Netherlands

**Keywords:** Anticoagulation, Vitamin K antagonists, Bridging, Heparin, Total hip arthroplasty, Total knee arthroplasty, Complications, Bleeding

## Abstract

**Background:**

Patients scheduled for elective surgery with a high risk of thromboembolism require anticoagulation bridging therapy perioperatively. The purpose of this study was to assess the risk of thromboembolic events and bleeding-related complications after total hip and knee arthroplasty in patients requiring bridging therapy for anticoagulants.

**Methods:**

A retrospective cohort study of all patients with primary total hip or total knee replacement in a 4-year period was performed. Outcome measures were blood loss, thromboembolic and bleeding-related complications and hospital stay.

**Results:**

Bridged patients had more blood loss and higher complication rates than the control group. Most complications were bleeding-related, and there were no thromboembolic events. Seven of the 14 (50 %) total hip patients bridged with unfractioned heparin required reoperation (three patients with ischial neuropraxia due to hematoma). There were two bleeding-related deaths in total hip patients bridged with low-molecular-weight heparin. Mean hospital stay was significantly longer in unfractioned heparin bridging.

**Conclusion:**

In this study, there was a significant increase in bleeding-related complications in total joint replacement with bridging therapy compared to prophylaxis. This risk was highest in patients with total hip arthroplasty. There were no thromboembolic events in bridged patients.

## Background

Patients with conditions causing a high risk for thromboembolism (atrial fibrillation, mechanical heart valves or recurrent venous thromboembolism) require long-term oral anticoagulation (OAC) therapy with vitamin K antagonists. Management of anticoagulation forms a challenge when these patients require elective surgery. Whereas interruption of OAC increases the risk of thromboembolic events, continuing results in a risk of bleeding-related complications. To balance these risks, OAC can be interrupted to obtain normal coagulation during surgery and restarted as soon as hemostasis is achieved. Based on the patient’s individual risk for thromboembolism, physicians determine whether bridging is required [1].

Given the long half-life of vitamin K antagonists, shorter acting anticoagulation agents such as unfractioned heparin (UFH) or low-molecular-weight heparin (LMWH) are used as bridging therapy. Traditionally, in-hospital bridging therapy with intravenously administered UFH has been used. The advantage is the monitoring ability with the activated partial thromboplastin time (APTT). Therapeutic LMWH has the potential benefit that outpatient treatment is possible. In recent prospective observational studies, LMWH was shown to be as safe as UFH in selective procedures with low thromboembolic event rates of 0.6–3.6 % and major bleeding rates of 0.5–11.3 % for both bridging therapies [2–5]. However, there was a non-significant trend towards an increased bleeding risk in patients undergoing major procedures in vascular and general surgery [2, 3]. Although over 40 % of the patients in these cohorts underwent major surgery, these studies pooled different procedures by surgical specialty and lacked control groups. Therefore, extrapolating this data to assess a patient’s individual complication risk for a specific procedure is impossible.

Total hip (THA) and total knee arthroplasty (TKA) are two of the most commonly performed major surgical procedures in orthopaedic surgery, with a high success rate. Without predisposing risk factors, the frequency of venous thromboembolisms after major orthopaedic surgery is high and most guidelines recommend the use of prophylaxis [6–9]. Despite prophylaxis, a small number of patients develops venous thromboembolism and requires a therapeutic dose of anticoagulation [7, 10, 11]. Previous studies [12–14] have shown a high incidence (up to 45 %) of bleeding-related complications and a considerable morbidity associated with a therapeutic dose anticoagulation started within 6 days after total joint arthroplasty (TJA).

The purpose of this study was to assess the risk of thromboembolic events and bleeding-related complications in patients undergoing TJA, who require bridging therapy perioperatively.

## Methods

In this retrospective cohort study, all patients receiving a primary THA or TKA in our tertiary referral centre for cardiac surgery were included in the years 2008–2012. The hospital ethics committee approved this study. All institutional patient records were reviewed by two authors (MH and DK). Medical records were used to obtain patient characteristics, date of surgery, blood loss, blood transfusion, complications and postoperative hospital stay. The electronic pharmacy database was used to search for pre- and postoperative anticoagulation therapy. The complication database was checked for complications within the first 3 months after surgery, and the Dutch National Orthopaedic Implant Registry (Landelijke Registratie Orthopedische Implantaten, LROI) was consulted. To minimize the risk of missing thromboembolic events, we matched the codes for THA and TKA with the codes for lower extremity ultrasound examination and CT-pulmonary-angiography. All radiology reports were checked.

The indication for bridging therapy was determined by the anesthesiologist, according to the Dutch National guidelines. In this guideline, the type of bridging therapy is not dictated. A multidisciplinary team consisting of the anesthesiologist, a cardiologist or lung specialist and the orthopaedic surgeon determined whether UFH or LMWH was prescribed, and the concerning physician was involved in the postoperative treatment of the patient during hospital stay. Dosage was adjusted for patient’s weight and renal function. Patients requiring bridging stopped with OAC 3–5 days before surgery. Patients on intravenous UFH were admitted to the hospital 3 days before surgery to start with treatment. Patients on LMWH (nadroparin subcutaneously in therapeutic dose) commenced treatment at home and were admitted the day before surgery. An international normalized ratio (INR) ≤1.5 was required for surgery. The target APTT ratio with UFH treatment was 2–2.5 and was measured 6-hourly. UFH was stopped 6 h before and restarted 12 h after surgery. OAC was restarted when there was no excessive wound draining. Bridging therapy was stopped at INR >2.0.

Platelet antiaggregants were stopped 5–10 days before surgery in all patients. In patients with OAC without indication for bridging, OAC was stopped 3–5 days before surgery and restarted after surgery when there was no excessive wound draining. Patients without bridging received a daily prophylactic dose nadroparin of 2850 international units (IU) (body weight <90 kg) or 5700 IU (body weight >90 kg). Prophylactic LMWH was initiated upon admission the evening before surgery and continued for 6 weeks postoperatively.

All THAs were performed with a standard posterolateral approach, and a standard medial parapatellar approach was used for TKAs. All TKAs were cemented. In all patients, a wound drain with vacuum was placed during surgery and removed after 24 h. No tranexamic acid or cell salvage was used.

The outcome measures were defined as blood loss, complications and duration of hospital stay. Parameters for blood loss were haemoglobin (Hb) decrease (preoperative value—lowest Hb level postoperatively) and transfusion requirements. Trigger points for transfusion were Hb ≤4.0 mmol/L in healthy patients (American Society of Anesthesiologists (ASA) class I), Hb ≤5.0 mmol/L in ASA II/III and Hb ≤6.0 mmol/L in ASA IV patients. The bleeding index was calculated (BI = units of packed cells + points Hb decrease) [15, 16]. The adverse events of interest were major and minor bleeding, neuropraxia due to hematoma, reoperation (such as evacuation of hematoma), arterial thromboembolism including cerebral vascular accidents (CVA), deep venous thrombosis (DVT), pulmonary embolism (PE), heparin-induced thrombocytopenia, infected TJA and death. Major bleeding was defined as a bleeding index >5.0 (more than 3 points of Hb decrease and transfusion with two or more packed cells) or need for operative evacuation of the haematoma. Minor bleedings were all other recorded bleeding events which may have resulted in wound healing problems or prolonged hospital stay. Readmittance within 3 months was also reported.

Statistical analysis was performed with SPSS software (version 21, IBM Corporation, Armonk, NY), and differences were considered significant at *p* < 0.05. Single factor analysis of variance (ANOVA) with Bonferroni (for equal variances) or Tamhane (for unequal variances) post hoc tests were used to compare the numerical values of the groups. Fisher’s exact test was used to compare the categorical variables of the groups.

## Results

Of the 2529 patients undergoing a THA or TKA in our hospital, 35 patients required bridging therapy for OAC. Indication for bridging therapy was a mechanical heart valve in 18 (14 aortic, two mitral, two both), biological heart valve in two, atrial fibrillation (AF) in three (CHADS2 score 5/6 and/or persistent AF), previous pulmonary embolism in six, hypercoagulopathy in three (one patient with Factor-V-Leiden and recurrent DVT and PE in combination with AF, one patient with hyperhomocysteinemia and protein C deficiency with previous DVT and one patient with hyperhomocysteinemia with recurrent DVT despite OAC), and an in-stent coronary stenosis in one patient. In one patient with DVT and PE after previous contralateral TKA and one patient with postoperative AF, immediate postoperative therapeutic LMWH was started. Although these patients did not receive OAC preoperatively, they were included in the LMWH bridging TKA group. Patient characteristics are listed in Table 1. There were no significant differences in mean age, gender or type of implant between the different groups. There was a significant difference in indication for TKA because of one patient with avascular necrosis in the LMWH group. Patients with a mechanical heart valve were more likely to receive UFH as bridging therapy (*p* < 0.001). As expected, OAC use was significantly higher and antiaggregants use significantly lower in bridging groups compared to the prophylaxis group.

**Table 1:** Total joint arthroplasty patient characteristics

	UFH bridging	LMWH bridging	LMWH prophylaxis	*p* value
	(*n* = 18)	(*n* = 17)	(*n* = 2494)	
Mean age (range)	73 (50–85)	72 (53–82)	70 (32–95)	0.222
Male/female	8/10	8/9	849/1645	0.348
Indication THA (*n* = 1517)				0.187
Osteoarthritis	10	6	1334	
Avascular necrosis	1	2	49	
Late posttraumatic	2	1	80	
Inflammatory arthritis	0	0	14	
Acute fracture	1	0	17	
Indication TKA (*n* = 1012)				<0.001
Osteoarthritis	4	7	975	
Avascular necrosis	0	1	2	
Late posttraumatic	0	0	7	
Inflammatory arthritis	0	0	16	
Procedure				0.932
Cemented TKA	4	8	1000	
Cemented THA	12	8	1233	
Uncemented THA	2	1	151	
Hybrid THA	0	0	30	
Resurfacing THA	0	0	80	
Indication for bridging therapy				<0.001
Mechanical heart valve	15	3		
Other reason	3	14		
Medication of interest				<0.001
Anticoagulation	16	13	151	
Antiaggregants	0	1^a^	482	
Combined	2	1	8	
None	0	2^b^	1853	

^a^Patient with in-stent coronary stenosis

^b^One patient with history of PE and one patient with AF de novo during operation

The 35 bridging patients had an average body mass index (BMI) of 27.54 (range 20.4–36.9, one patient unknown), diabetes was present in seven patients (three unknown) and the average ASA score was 2.47 (one unknown). Of the bridged patients, four actively smoked, four had a past of smoking and three were unknown. The average operating time including anaesthesia time of these patients was 87 min.

The patients that were bridged had a significant higher complication rate and significantly more bleeding-related complications. For UFH, the OR was 19.8 (95 % CI 6.50–60.60, *p* < 0.0001) and 36.8 (95 % CI 14.14–95.59, *p* < 0.0001), respectively. For LMWH bridging, the OR was 6.4 (95 % CI 2.44–16.63, *p* = 0.0002) and 9.1 (95 % CI 2.89–28.36, *p* = 0.0002), respectively. Comparison between THA and TKA patients did not render statistical significant results.

### Total hip arthroplasty

Of the 1517 patients undergoing THA, 23 required bridging therapy. Table 2 summarizes the outcome measures after THA. Although there were no major outliers in APTT, patients bridged with UFH had significantly higher decreases of haemoglobin levels, number of transfused units and bleeding index compared to patients on prophylaxis. There were no significant differences in haemoglobin levels, number of transfused units and bleeding index between the therapeutic and prophylactic LMWH groups. Mean hospital stay for patients with UFH was significantly longer (*p* = 0.009) compared to patients on LMWH bridging therapy or prophylaxis, even after compensation for the 3 days of preoperative admission.

**Table 2:** Blood loss, length of hospital stay and complications after total hip arthroplasty

		UFH bridging	LMWH bridging	LMWH prophylaxis	*p* value
		(*n* = 14)	(*n* = 9)	(*n* = 1494)	
Blood loss					
Preop. haemoglobin level, mmol/L (range)		8.0 (6.1–9.4)	8.2 (5.9–9.6)	8.5 (5.3–11.1)^a^	0.313
Postop. haemoglobin level, mmol/L (range)		5.0 (3.2–6.8)	6.0 (3.7–8.2)	6.5 (3.6–9.0)^b^	<0.001
Haemoglobin decrease, mmol/L (range)		3.0 (0.4–5.3)	2.2 (0.3–5.2)	2.0 (0.3–5.3)^c^	0.044
Transfusion units	Mean (range)	5.8 (0–25)	2.9 (0–18)	0.2 (0–6)	0.040
	Median	4	0	0	
Bleeding index	Mean (range)	8.8 (0.4–30)	5.0 (0.3–22)	2.1 (0.3–9.3)^c^	0.027
	Median	6.9	2.2	1.9	
Hospital stay					
	Mean days (range)	23.9 (7–71)	9.9 (4–27)	6.8 (4–69)	0.009
	Median	16	8	6	
Complications					
Any complication		11 (79 %)	4 (44 %)	218 (14.6 %)	<0.001
Bleeding-related complication					
Major bleeding		9 (64 %)	3 (33 %)	46 (3.1 %)	<0.001
Minor bleeding		0	0	5 (0.3 %)	1.000
Neuropraxia due to hematoma		3 (21 %)	1 (11 %)	0	<0.001
Evacuation of hematoma		7 (50 %)	1 (11 %)	3 (0.2 %)	<0.001
Thromboembolic complication					
Arterial thromboembolism		0	0	4 (0.3 %)	1.000
Deep venous thrombosis		0	0	7 (0.5 %)	1.000
Pulmonary embolism		0	0	3 (0.2 %)	1.000
Heparin-induced thrombocytopenia		1 (7 %)	0	0	NA^d^
Infected THA		1 (7 %)	0	13 (0.9 %)	0.194
Death		0	2 (22 %)	4 (0.3 %)	0.001

^a^Twenty-six values unknown

^b^Four values unknown

^c^Twenty-eight values cannot be calculated

^d^Not applicable, number too small to perform statistics

Bridging patients were significantly more at risk to have adverse events compared to the prophylaxis group. Fifteen of the 23 patients (65 %) on bridging therapy had one or more complications (OR 10.9, 95% CI 5.36–22.02, *p* < 0.0001). The complications were bleeding-related in 10 out of 11 for UFH and three out of four for LMWH. Bleeding-related complications were significantly higher in THA patients with bridging therapy (odds ratio of 50.9 for UFH and 14.5 for LMWH versus prophylaxis). Major bleeding occurred in, respectively, nine (64 %) and three (33 %) of the UFH and LMWH patients. In eight bridging patients (seven on UFH and one on LMWH), reoperation with evacuation of the hematoma was required. In four (three on UFH and one on LMWH), this was indicated by neuropraxia of the ischial nerve which developed in the first postoperative days. There were no thromboembolic events in patients on bridging therapy. In the control group, nine patients (0.6 %) developed a venous thromboembolism (six DVT, two PE, one both) despite prophylactic LMWH. One had temporarily stopped OAC which was indicated for paroxysmal AF. Other patients with thromboembolism did not use OAC preoperatively. The earliest DVT or PE in the prophylaxis group occurred 3 days postoperatively. No adverse events occurred after initiation of OAC as treatment for the thromboembolism in these patients. Four patients in the prophylaxis group had an arterial thromboembolism. Two patients were on temporarily stopped antiaggregants and developed a myocardial infarction. OAC or antiaggregants were also temporarily stopped in two patients with an ischemic CVA.

Thirteen patients on prophylactic LMWH (0.9 %) had an early THA infection which was treated with open debridement, antibiotics and implant retention. Deep infection developed in none of the LMWH bridging patients and in one of the UFH bridging patients (7 %). Although the patient on UFH bridging therapy was also treated with open debridement and antibiotics, the multiresistant *Escherichia coli* failed to respond and a temporary Girdlestone was created with vacuum-assisted wound closure to treat the infection. Eventually, the patient underwent uncomplicated implantation of THA with LMWH bridging therapy in a tertiary-care infectious disease and orthopaedic centre. In the first 3 months postoperative, there were two deaths (22 %) in the LMWH bridging group (massive abdominal bleeding; rectus hematoma with abdominal compartment syndrome and acute renal failure) and four deaths (0.3 %) in the prophylaxis group (sepsis due to early THA infection; sepsis possibly due to endocarditis; intracerebral haemorrhage and herniation syndrome due to undiagnosed brain tumour; out of hospital death of unknown cause 14 days postoperatively). None of the UFH patients died in the first 3 months.

### Total knee arthroplasty

Twelve of 1012 patients undergoing TKA required bridging therapy. Blood loss, complications and hospital stay are summarized in Table 3. Although there were no major outliers in APTT, patients on UFH bridging therapy undergoing TKA had a significant higher decrease of haemoglobin levels and higher bleeding index compared to patients on prophylaxis. There were no significant differences in blood loss between the therapeutic and prophylactic LMWH groups. The mean hospital stay was not significantly different between the groups.

**Table 3:** Blood loss, length of hospital stay and complications after total knee arthroplasty

		UFH bridging	LMWH bridging	LMWH prophylaxis	*p* value
		(*n* = 4)	(*n* = 8)	(*n* = 1000)	
Blood loss					
Preop. haemoglobin level, mmol/L (range)		8.5 (8.0–9.2)	8.6 (7.6–10.3)	8.5 (6.2–10.9)^a^	0.902
Postop. haemoglobin level, mmol/L (range)		5.6 (4.9–6.0)	6.4 (5.0–9.0)	6.5 (4.0–9.9)^b^	0.104
Haemoglobin decrease, mmol/L (range)		3.0 (2.1–3.9)	2.2 (1.3–4.2)	2.0 (0.2–4.8)^c^	0.012
Transfusion units	*n* (range)	0.5 (0–2)	0 (0)	0.15 (0–9)	0.449
	Median	0.0	0.0	0.0	
Bleeding index	Mean (range)	3.5 (2.1–5.9)	2.2 (1.3–4.2)	2.1 (0.2–12.4)^c^	0.045
	Median	2.9	1.9	2.0	
Hospital stay					
	Mean days (range)	32.5 (12–83)	9.6 (5–20)	7.4 (4–112)	0.552
	Median	17.5	8.5	7	
Complications					
Any complication		3 (75 %)	5 (63 %)	156 (15.6 %)	<0.001
Bleeding-related complication					
Major bleeding		1 (25 %)	1 (13 %)	26 (2.6 %)	0.041
Minor bleeding		2 (50 %)	1 (13 %)	5 (0.5 %)	<0.001
Neuropraxia due to hematoma		0	0	0	NA^d^
Evacuation of hematoma		0	0	1 (0.1 %)	NA^d^
Thromboembolic complication					
Arterial thromboembolism		0	0	9 (0.9 %)	1.000
Deep venous thrombosis		0	0	13 (1.3 %)	1.000
Pulmonary embolism		0	0	5 (0.5 %)	1.000
Heparin-induced thrombocytopenia		0	0	0	NA^d^
Infected TKA		1 (25 %)	1 (13 %)	8 (0.8 %)	0.006
Death		0	0	4 (0.4 %)	1.000

^a^Twenty-two values unknown

^b^Three values unknown

^c^Twenty-five values cannot be calculated

^d^Not applicable, number too small to perform statistics

TKA patients on bridging therapy were more likely to have adverse events compared to the prophylaxis group. Eight of the 12 patients (67 %) on bridging therapy had one or more complications (OR 10.8, 95% CI 3.22–36.37, *p* < 0.0001). In two out of five patients bridged with LMWH and all three patients bridged with UFH, the complications were bleeding-related (OR 93.8 for UFH and 10.4 for LMWH bridging). There were two major and three minor bleedings in patients on bridging therapy for which admission was prolonged. No thromboembolic events occurred in patients on bridging therapy. In the patients on prophylaxis, nine arterial thromboembolic events, 13 DVTs and five PEs occurred. The arterial thromboembolic events consisted of two patients with myocardial infarction postoperatively after temporarily stopped antiaggregants, a patient with transient ischaemic attack and three patients with ischaemic CVA. One on interrupted antiaggregants and one on interrupted OAC. The other patient did not receive preoperative OAC or antiaggregants and died of CVA in the brain stem. In two patients with temporarily stopped antiaggregants, an embolectomy was required for arterial occlusions at the trifurcation of the popliteal artery. These two patients also had DVT. Of the other patients with DVT, OAC and antiaggregants were temporarily stopped in three and two patients, respectively. None of the patients with pulmonary embolisms were on preoperative OAC or antiaggregants.

Of the 10 patients (1 %) with an early deep TKA infection, one (25 %) was on UFH and one (13 %) was on therapeutic LMWH. All patients with an early infected TKA were treated with open debridement, antibiotics and retention of implant. One of the patients on prophylaxis developed wound dehiscence after falling, complicated by implant infection and sepsis. This patient had a history of liver cirrhosis, ascites and congestive heart failure and died 5 months after TKA of causes related to complications. Three other prophylaxis patients died in the first days after TKA (two sudden deaths of unknown cause; one brain stem CVA).

## Discussion

Managing complication risks in patients requiring bridging therapy during TJA remains a challenge. In this retrospective study, patients on bridging therapy had a significantly higher complication risk compared to patients receiving standard thrombosis prophylaxis. Among 35 patients requiring bridging therapy, 66 % had a complication in the first 3 months after TJA. The majority of the complications were bleeding-related, and there were no thromboembolic events in both bridging groups. Comparing both bridging groups did not render statistically significant results for (bleeding-related) complications. There were differences in odds ratios between the groups, however. Patients bridged with UFH had the highest risk of bleeding-related complications in TJA. In TKA patients, the odds ratio for bridging with UFH was 93.8, compared to 10.4 for LMWH. In THA patients, this differe nce in odds ratios was smaller (50.9 for UFH compared to 35.4. for LMWH). For the THA patients, there were major consequences like neuropraxia of the ischial nerve or reoperation. Although no bridging therapy was required according to the guideline of the American College of Chest Physicians (ACCP) [7], six of the 151 patients on interrupted OAC (4 %) received normal prophylactic LMWH dose and still developed thromboembolic events.

The bleeding-related complication rate of 40 % in this study is much higher than general reported rates in elective surgery of 0.9–11.3 % [3–5, 17–23]. However, it appears not to be incidental as similar high rates are reported in previous studies of perioperative warfarin therapy in TKA and THA [14, 24]. Although patients without bridging therapy and with therapeutic UFH or LMWH therapy were pooled in these studies, the overall rate of excessive wound drainage and superficial or deep infections were significantly higher in patients on warfarin therapy compared to a control group. Similar to our results, the complication rates were highest in a subgroup of patients on intravenous UFH bridging with 44 and 53 % of the patients developing excessive wound drainage, 15 and 28 % developing a superficial wound infection and 11 and 19 % developing deep infection after THA and TKA, respectively. In our study, TKA bridging patients developed less neuropraxia and required less reoperations compared to THA, possibly due to the fact that the knee is an encapsulated compartment. The rising of pressure inside the compartment and the appliance of a pressure bandage around the knee may aid coagulation.

Considering the high number of bleeding-related complications and low thromboembolic rates in patients on bridging therapy, it appears that the risks are not well balanced. Although no thromboembolic events occurred in patients on bridging therapy, the mortality and morbidity of these complications is significant. This might explain a tendency to over-coagulate these patients. For patients with mechanical heart valves on oral anticoagulation therapy, the incidence of valve thrombosis is 0.5 to 6 % per patient-year for aorta and mitralis positions, respectively [25–27]. For patients with a history of DVT or pulmonary embolism, the long-term complication risk is also significant. After 3 months of treatment with oral anticoagulation, the incidence of recurrent thromboembolism can be as high as 30 % within 8 years [28]. Twenty percent of these recurrent thromboembolic events were pulmonary embolisms, which were fatal in more than 50 % of the cases. In addition to the higher risk of recurrent events, these patients also have considerable risk of developing a postthrombotic syndrome or chronic thromboembolic pulmonary hypertension.

There is also a tendency of over-treating low-risk patients, as a recent study reported a poor adherence to guidelines [29]. In this study, 84.3 % of the patients received LMWH bridging despite a low thromboembolic risk profile and had high major bleeding rates. In our bridging groups, all patients had a high thromboembolic risk; therefore, the indication for bridging according to ACCP guidelines was correct and no over-treated patients were detected.

Just as thromboembolism, increased anticoagulated state may also cause significant morbidity and mortality. Obviously, the increased mortality rate due to bleeding-related complications of two out of 35 patients on bridging therapy (5.7 %) compared to seven out of 2494 control patients (0.3 %) is striking. The higher rate of deep infections and neuropraxia of the ischial nerve causes long-term impairment. In this study, the majority of the complications were excessive wound drainage and local major bleedings which do not have detrimental effects on long-term morbidity.

Timing of initiation of the postoperative bridging has been suggested as a critical factor for complications [13, 14, 24]. In 112 patients requiring therapeutic anticoagulation for thromboembolic events in the early postoperative phase, the complication rate was 45 % when UFH started within 6 days after TJA and dropped to 15 % when UFH was started more than 6 days postoperatively [13]. However, a smaller study with 29 patients was not able to show differences in bleeding-related complications when therapeutic dosage of anticoagulation was started in the first 2 days postoperatively or later [14]. A 6-day time frame is not clinically relevant for patients on bridging therapy, since therapy needs to be restarted on the day of surgery. Despite the risks of early initiation of the bridging therapy, our overall complication rate was similar to the previous reported rates of starting anticoagulation within 6 days postoperatively [13, 14].

Two severe complications were the two deaths in the LMWH bridging group. Both patients died of bleeding-related complications. These complications were not directly related to joint replacement surgery, but to spontaneous bleeding elsewhere. One patient, a 73-year-old woman with an ASA score of 2, developed a big abdominal rectus muscle haematoma with an abdominal compartment syndrome. The lowest recorded postoperative Hb was 4.1 mmol/L, and in total, there was a required transfusion of 18 packed cells. The patient was admitted to the ICU and developed acute renal failure. Further treatment was ceased based on her own and direct family’s wishes. The family did not give permission for section of the body. The other patient was an 82-year-old woman with an ASA score of 3. The day after surgery, the Hb was 5.2 mmol/L, were after, two packed cells were administered. The patient collapsed on the ward days after surgery and resuscitation was unsuccessful. Section of the body showed approximately 1 L of old blood in the small intestine. These two deaths probably are the result of spontaneous bleeding due to anticoagulation. This emphasizes the serious risk of bridging therapy. Although both patients were bridged with LMWH, the data shows the risks with UFH are just as high, if not higher.

Although the data of this study is retrospective, the search of multiple sources was comprehensive. In contrast to the matched control groups in previous studies, all patients receiving a TJA were included in this study [14, 24]. Despite the large control group, one of the limitations is the small group size of bridging patients. Comparing both bridging groups did not render significant results. Given the nature of this retrospective study, there is also the risk of selection bias for type of bridging. Based on this data, it is difficult to recommend one of the two bridging protocols. Given the higher odds ratios for any complication and bleeding-related complications with UFH bridging, the advantage of outpatient treatment with LMWH and thus shorter hospital admittance, we now opt for LMWH bridging in our department.

## Conclusions

In conclusion, in this study, bridging oral anticoagulants with UFH or LMWH during total joint replacement was safe from a thromboembolic perspective. However, there was a significant increase in bleeding-related complications with bridging therapy, with serious consequences for the patients. This risk was the highest in patients with total hip arthroplasty. The risks of bleeding-related and thromboembolic complications have to be carefully balanced. The high bleeding-related complication rate in patients on bridging therapy warrants counselling of patients preoperatively and careful monitoring postoperatively. This data also highlights the need for future studies, including randomized trials, to compare the safety of UFH and LMWH bridging therapy in patients undergoing TJA.

## Abbreviations

ACCP: American College of Chest Physicians
AF: atrial fibrillation
APTT: activated partial thromboplastin time
ASA: American Society of Anesthesiologists
BMI: body mass index
CVA: cerebral vascular accidents
DVT: deep venous thrombosis
Hb: haemoglobin
INR: international normalized ratio
IU: international units
LMWH: low-molecular-weight heparin
OAC: oral anticoagulation
PE: pulmonary embolism
THA: total hip arthroplasty
TJA: total joint arthroplasty
TKA: total knee arthroplasty
UFH: unfractioned heparin
